# Assessment of Vaccination Impact in PPR-Control Program Implemented in Southern States of India: A System Dynamics Model Approach

**DOI:** 10.3390/v17010023

**Published:** 2024-12-27

**Authors:** Govindaraj Gurrappanaidu, Naveen Kumar Gajalavarahalli Subbanna, Francis Wanyoike, Sirak Bahta, Yeswanth Raghuram Reddy, Dwaipayan Bardhan, Balamurugan Vinayagamurthy, Kennady Vijayalakshmy, Rahman Habibur

**Affiliations:** 1 ICAR-National Institute of Veterinary Epidemiology and Disease Informatics, Post Box 6450, Yelahanka, Bengaluru 560064, India; gsnaveenkumar93@gmail.com (N.K.G.S.); raghuramreddyyeswanth@gmail.com (Y.R.R.); balavirol@gmail.com (B.V.); 2 International Livestock Research Institute (ILRI), Post Box 30709, Nairobi 00100, Kenya; f.wanyoike@cgiar.org (F.W.); s.bahta@cgiar.org (S.B.); 3 ICAR-Indian Veterinary Research Institute, Bareilly 243122, India; dwaipayanbardhan@gmail.com; 4 International Livestock Research Institute (ILRI), Block-C, First Floor, NASC Complex, CG Centre, DPS Marg, Pusa, New Delhi 110012, India; v.kennady@cgiar.org (K.V.); r.habibar@cgiar.org (R.H.)

**Keywords:** PPR, vaccination impact, SD modeling

## Abstract

Mass vaccination against peste des petits ruminants (PPR) in two southern states of India, namely Andhra Pradesh and Karnataka, has reduced disease outbreaks significantly. The sporadic outbreaks reported now can be attributed in part to the recurring movement of sheep and goats between these contiguous states. This study assessed the present level of economic burden and impact of vaccination on the local system (one state), considering the exposure from the external system (neighboring state) using a system dynamic (SD) model. The SD model relies on interdependence, interaction, information feedback, and circular causality and captures potential feedback between disease control interventions and their impact on various epidemiological and economic outcomes. The data for parameterization of the model were collected through surveys, expert elicitation, and literature review. The sporadic outbreaks reported in recent years (<10 outbreaks/year during 2022) were due to continuous “mass vaccination” for more than a decade. During 2021–2022, the PPR incidence was less in both the states, with an estimated loss of USD 26.30 and USD 22.86 million in Andhra Pradesh and Karnataka, respectively. The SD model results showed a systemic increase in flock size and offtakes and a decline in the number of infected and death cases under high vaccination coverage (75% and 100% coverage) compared to the low-coverage scenario. Hence, the coordinated inter-state vaccination efforts offer better prospects, as efforts in one state have positive externalities in terms of fewer outbreaks in a neighboring state.

## 1. Introduction

Peste des petits ruminants (PPR) is a highly contagious and economically important viral disease in sheep and goats caused by PPR virus, which belongs to the *Morbillivirus Caprinae* species under genus *Morbillivirus* of the family *Paramyxoviridae.* The symptoms of the disease include severe pyrexia, mucopurulent oculo-nasal discharges, necrotizing stomatitis, enteritis, and pneumonia [1]. The disease causes high mortality and morbidity in naïve flocks, and >50% of deaths were reported in affected naïve flocks [2,3]. PPR was first reported in sheep and goats in 1942 in Côte d’Ivoire and in India it was reported in Tamil Nadu in 1987 [4,5]. The disease was reported only in the southern states of India until 1994, and later, it spread to other states and became enzootic in the country [1,6]. High mortality and morbidity due to PPR caused significant economic burden to various stakeholders associated with the small ruminant value chain. In India, the estimated annual loss reported in the literature was INR 88,951 million [7], INR 16,116 million [3], and INR 45,710–46,830 million (USD 653–669 million) [8]. To control the disease, a vero cell line-based live attenuated PPR vaccine that provides immunity for 3 to 6 years was developed [9,10].

In India, “focused vaccination” against PPR was practiced in the locations of the outbreak from 2003–2004 to 2009–2010 (In India, financial year refers to April to March of next year i.e., April 2003 to March 2004 is written as 2003-04); The calendar years refers to January to December and it is written as 2003 for one year and 2003–2004 for two calendar years). Later, since 2010–2011, a “mass vaccination programme” was implemented through the Government of India’s PPR-control program (PPR-CP) [4]. Under PPR-CP, mass vaccination with 100 percent coverage of the target population was practiced during the first year, followed by bi-annual vaccination for the next two years to cover the unvaccinated and naïve sheep and goat population [11]. To date, control strategies have been mainly based on annual national mass vaccination campaigns and/or focused vaccination in response to overt outbreaks. Mass annual vaccination is an effective control measure, but in practice, it is difficult to achieve and costly [12].

Among the southern states that implemented PPR-CP, Karnataka and Andhra Pradesh adopted a mass vaccination strategy. In Karnataka, the disease was first reported in 1992 [13] and spread across the state, and peak outbreaks were reported during 2004–2006. To contain PPR, the state government adopted focused vaccination from 2003 to 2010 using an experimental batch of the vaccine. After implementation of the PPR-CP in 2010, the state adopted a mass vaccination program that resulted in a significant reduction in outbreaks (from 184 outbreaks during 2004–2005 to 8 outbreaks during 2012–2013) [11,12]. Similar to Karnataka, the neighboring state of Andhra Pradesh also reported 183 PPR outbreaks during 1998, and it peaked at 532 outbreaks in 2004–2005 and declined thereafter [11]. In 2005, a loss of INR 1265 million was estimated in the state [14]. Andhra Pradesh implemented mass vaccination programs from January 2007 to March 2008, covering 35 million sheep and goats, followed by annual vaccination campaigns until 2010 [11,14]. The initial success has prompted the state government to continue mass vaccination campaigns as a part of India’s government-sponsored PPR-CP since 2010, resulting in a significant decline in outbreaks (<10 outbreaks during the calendar year 2021–2022 [14]. The success of the program in these states was mainly due to the availability of vaccines, financial and policy support from the state and the central government, concerted efforts from field veterinarians, and the active participation of farmers in the vaccination campaigns [11,15].

Though there was a significant decline in outbreaks after mass vaccination implementation, these states still report sporadic PPR outbreaks. This can be attributed, in part, to the recurring movement of sheep and goats between contiguous states like Karnataka and Andhra Pradesh and also with the other contiguous neighboring states for grazing, trade, and transit, thus leading to a perpetual exposure of animals within Karnataka and Andhra Pradesh to the PPR virus. This two-way interaction between the neighboring states with varied levels of vaccination and differences in vaccination timings and coverage implies inter-dependency in disease occurrence and its management. Any negligence in PPR vaccination at home state and in neighboring states could potentially impact the health of sheep and goats. Given this intricate interplay, to comprehensively understand the multifaceted impact of the PPR burden in the local system (Karnataka) with the changes in vaccination coverage within the state and with a minimum influence of disease from the external system (from the neighboring state, Andhra Pradesh) and vice versa, a system dynamics (SD) model encompassing flock dynamics, disease transmission, vaccination strategies, and marketing dynamics was utilized in this study.

## 2. Materials and Methods

### 2.1. Study Area

The study was undertaken in Andhra Pradesh and Karnataka, as they are contiguous states. The state of Karnataka is the sixth-largest state, comprising 31 districts with an area of 191,791 km^2^, i.e., 5.83 per cent of India’s total geographical area. Karnataka is the only southern state to have land borders with all of the other four southern Indian sister states. It is bordered by the Arabian Sea to the west, Goa to the northwest, Maharashtra to the north, Telangana to the northeast, Andhra Pradesh to the east, Tamil Nadu to the southeast, and Kerala to the south. As per the 20th livestock census in 2019, the total livestock population in the state is 29.0 million, of which sheep and goats constitute 17.21 million (11.05 million sheep and 6.16 million goats). The Mandya District is located in southern part of the state, with a sheep and goat population of 0.6 million, and Bellary District is located in central eastern part, with a population of 1.1 million sheep and goats.

Andhra Pradesh is the seventh-largest state in India with an area of 162,968 km^2^. The state is bordered by Tamil Nadu in the south, in the east by the Bay of Bengal Sea, in the northeast by Odisha, in the north and northwest by Telangana, and in the west and southwest by Karnataka. As per the 20th livestock census in 2019, the total livestock population in Andhra Pradesh state is 34.0 million, of which sheep and goats constitute 23.12 million (17.6 million sheep and 5.52 million goats). The Ananthapur District is located in southwest part of the state, with a sheep and goat population of 6.8 million, and Prakasam District is located in central-eastern part, with a sheep and goat population of 1.3 million sheep and goats.

### 2.2. Data Collection

The secondary data on PPR outbreaks, attacks, and death cases reported in Karnataka and Andhra Pradesh were collected and analyzed to understand the PPR disease occurrence pattern from 2008 to 2022. Further, the primary data on the socio-economic parameters of sheep and goat inventory; production parameters; disease incidence, mortality, and morbidity across species, age, and sex; and vaccination details, including cost incurred, treatment cost, and opportunity cost of labor, were collected from the sheep- and goat-rearing farmers in the surveyed villages. Besides farmers, the data on PPR incidence, management pattern, and cost associated with disease treatment were collected from animal traders, and prices of meat and quantity of meat sold per day were collected from the retail meat sellers. The data required for parameterization of the SD model were collected from the PPR experts (scientists associated with PPR research, disease diagnosis, surveillance, and monitoring and field veterinarian) and literature review. The details of the collected variables and their source are provided in the Supplementary Table S1.

### 2.3. Sample Size

The sample size for the primary survey was calculated based on [16]:

$$SS=\frac{Z^{2}\left(P\right)\left(1-P\right)}{e^{2}}$$

where *SS* is the required sample size; *Z* is the Z-value at 95% confidence interval (1.96); P is the proportion of sheep and goat rearing households to the total number of households rearing livestock in rural areas (Andhra Pradesh (0.188) and Karnataka (0.225)); *e* is the acceptable sampling error (5%).

The estimated total sample size was 267 and 234 sheep- and goat-rearing flocks in Karnataka and Andhra Pradesh, respectively. However, 290 and 267 flocks from Karnataka and Andhra Pradesh were surveyed.

### 2.4. Sampling Procedure

A cross-sectional survey among sheep- and goat-rearing households in Karnataka and Andhra Pradesh was undertaken during 2020 and 2021. A multistage, random sampling procedure was followed to conduct the primary survey. In the first stage, the districts of states were grouped into two quartiles (high-PPR-risk and low-PPR-risk districts) based on sheep and goat population density (km^2^) and reported PPR outbreaks in the three years preceding the survey. In the second stage, one district from each of the high- and low-risk quartiles was randomly selected. In the third stage, in each of the selected districts, three to four blocks were randomly selected. In the fourth stage, in each selected block, one veterinary dispensary and 5–10 villages under this dispensary were selected randomly. In the last stage, the individual farmers were selected randomly in the identified villages and interviewed using a pre-tested schedule. The distribution of the sample farmers was proportional to the sheep- and goat-rearing households (obtained from the jurisdictional veterinarian) in the identified villages. Besides farmers, the data PPR from the sheep and goat traders and retailers in the respective survey locations were also collected.

### 2.5. Identification of PPR-Affected Farms

Among the surveyed farms, photographs of various clinical signs of PPR [4] were provided to the farmers to identify PPR infection in their flock. Based on farmers’ observation of PPR clinical symptoms, the surveyed flocks were grouped into PPR-affected and not-affected. Further, the PPR incidence in the flocks was triangulated with the field veterinarian for confirmation.

### 2.6. Epidemiological Parametres

The number of animals at PPR risk, those affected by PPR, and animals that died was collected during the survey, and the disease incidence and case fatality rate (CFR) across species, sex, and age groups were calculated.

### 2.7. Estimation of Per-Animal Loss

The sheep and goat inventory, clinically diagnosed PPR cases, and deaths were considered to estimate the primary metrics of mortality and morbidity levels using the deterministic models. The mortality loss was estimated based on the number of animals that died in each age and sex group, multiplied by the market value of the apparently healthy animals. The weight loss was assessed based on an average weight reduction (10% weight reduction) [17] in the PPR-infected animals, multiplied by live weight price (kg). The distress sale loss was calculated based on the number of animals sold under distress, multiplied by the price difference (actual price during the healthy state minus the distress sale value of the animals). The treatment cost was calculated based on the expenditure made for drugs/medicines, veterinarian charges, and transaction cost during the PPR outbreak in the flock and converted to per animal cost. Similarly, the opportunity cost of labor was calculated based on the incremental labor hours spent by the farm family members or hired members, multiplied by the prevailing wage rate. After calculating the loss per flock, appropriate weights were considered based on the number of animals that died, were infected, and recovered among the sheep and goats to calculate the per-animal loss. The market value/price of animals of various age groups and the labor wage rate that prevailed in the surveyed villages during the survey period (2020 and 2021) were considered for estimating the various losses. The estimated disease cost per animal was converted at the constant price for the years 2022, based on the consumer price index that prevailed during the respective years.

### 2.8. System Dynamics Model (SD)

The SD model approach is applied to dynamic problems that arise from complex social, managerial, economic, or ecological systems [18,19]. This model employs computer simulation on a parameterized model to test hypotheses about the relationship between the system structure and behavior. Quantitative SD models are systems of nonlinear differential equations that specify behavior and relationships in complex systems [20]. However, rather than representing such mathematical complexity through programming code, SD uses graphical features that allow for easier comprehension and interpretation.

To comprehensively understand the multifaceted impact of various levels of vaccination in Karnataka with the minimum disease influence from the external system (Andhra Pradesh) and vice versa, a system dynamics (SD) model encompassing flock dynamics, disease transmission, vaccination strategies, and marketing dynamics was attempted in this study. Both the states initiated the vaccination program a decade ago. Hence, a very high level of vaccination coverage (73 to 77%) and less PPR incidence (<4%) is now observed in these states. The probable reason for the sporadic incidence of PPR, in part, may be attributed to movement of the sheep/goats between these contiguous states. The study intended to understand, if vaccination coverage declines/or changes in a state (local system), what the impact (in the local system) will be with a given fixed minimum influence of the disease (assumed 5%) from the external system, mainly due to movement of animals between these two systems. The SD model was used to assess the PPR vaccination impact on PPR incidence, mortality, offtake, and earnings, considering the interplay of various variables associated with PPR incidence.

### 2.9. Statistical Analysis

Descriptive statistics, chi-square test, and a deterministic model to estimate the farm-level loss per animal were carried out using an Excel spreadsheet, and the state-level vaccination impact was assessed using a system dynamic (SD) model using Stella architect version 2.1.1. (address: 24 Hanover St, Ste 8A Lebanon NH 03766, USA).

## 3. Results

### 3.1. PPR Outbreaks, Diagnosed Cases, and Death Cases Trend in Karnataka and Andhra Pradesh

**Figure 1:**
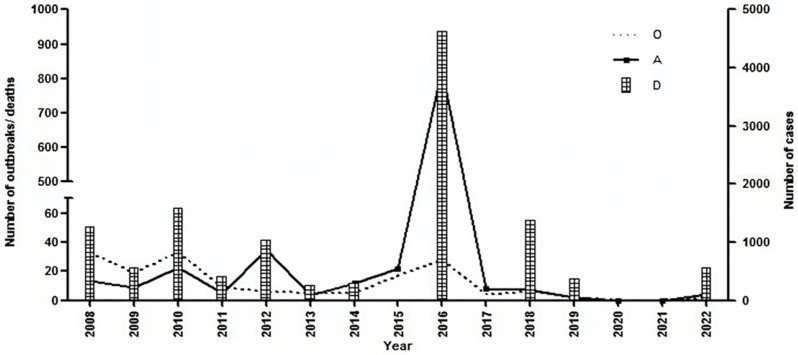
Number of PPR outbreaks, diagnosed cases, and death cases reported in Karnataka during 2008 to 2022.

**Figure 2:**
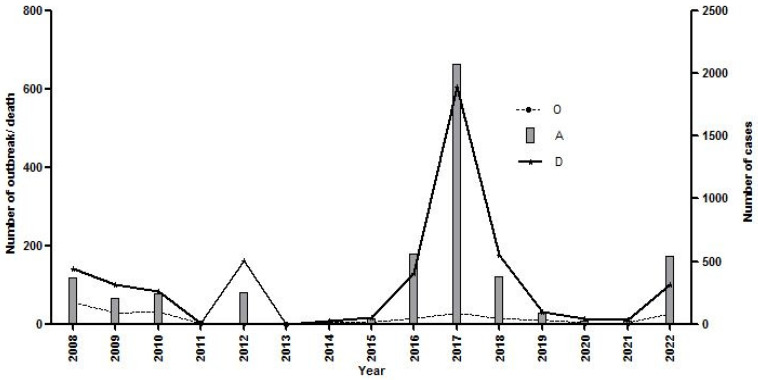
Number of PPR outbreaks, diagnosed cases, and death cases reported in Andhra Pradesh during 2008–2022.

In Karnataka, before mass vaccination, the number of outbreaks/year was >150 [11,12], but after mass vaccination implementation, the number of outbreaks/year declined to <10 during 2011–2015 and 2017–2022, except for 17 outbreaks (542 attacks and 124 death cases) during 2015 and 28 outbreaks (3961 attacks and 937 death cases) during 2016 (Figure 1). Similar to Karnataka, Andhra Pradesh also reported 183 PPR outbreaks during 1998, and it peaked at 532 outbreaks in 2004–2005 [11]; after the implementation of mass vaccination in 2007, the number of outbreaks declined considerably (Figure 2).

### 3.2. Socio-Economic Characteristics of Sample Flocks

The majority of the sheep- and goat-rearing farmers had limited literacy opportunities and did not have secure land tenure. The landless and small farmers comprised 92.1% of farmers in Andhra Pradesh and 87.2% in Karnataka. The annual farm income of 43% and 48% of the sheep- and goat-rearing farmers in Andhra Pradesh and Karnataka, respectively, was less than USD < 1250. The average flock size was 123 and 94 animals in Andhra Pradesh and Karnataka, respectively, with considerable variation between the study districts (Table 1).

### 3.3. PPR Incidence, Death Cases, and Case Fatality Rate (CFR)

In sheep, the PPR incidence in the survey period was 3.3% and 3.8%, and mortality amounted to 2.7% and 2.5% in the states of Andhra Pradesh and Karnataka, respectively. A significant difference in PPR incidence was observed between <6-months, 6–12-months, and >1-year age groups in Andhra Pradesh (χ^2^ = 1574; *p* < 0.01) and Karnataka (χ^2^ = 464; *p* < 0.01). A significant difference in PPR death cases was observed across age groups in Andhra Pradesh (χ^2^ = 1427; *p* < 0.01) and Karnataka (χ^2^ = 837; *p* < 0.01). Similarly, a significant difference in PPR incidence and death cases was observed between male and female sheep in both states. The CFR was considerably higher in animals <6 months of age than in the older animals (Table 2).

In goats, the PPR incidence was 0.8% and 1.2%, and the mortality level was 0.8% and 0.6% in Andhra Pradesh and Karnataka, respectively. A significant difference in PPR incidence among various age groups was observed in Andhra Pradesh (χ^2^ = 14; *p* < 0.01) and Karnataka (χ^2^ = 36; *p* < 0.01). The PPR death cases also showed a significant difference between the age groups in Andhra Pradesh (χ^2^ = 14; *p* < 0.01) and Karnataka (χ^2^ = 36; *p* < 0.01), but no significant difference was observed between the different sex groups (Table 2). The CFR was considerably higher in younger goats in both the states.

### 3.4. Estimated Loss per Animal

The pooled results showed that the mortality loss, body weight reduction loss, distress sale loss, treatment cost, and the opportunity cost of labor per animal were within the ranges of USD 35.1–108.1 and USD 40.5–189.2; USD 3.4–11.5 and 4.1–16.2; USD 60.8–114.9 and 40.5–103.6; USD 0.3–4.5 and USD 0.3–3.4; and USD 0.2–5.1 and 0.2–3.0 in Andhra Pradesh and Karnataka states, respectively. A detailed estimate of different losses in sheep and goats is presented in Table 3.

### 3.5. Projected Loss

The projected loss due to PPR in Andhra Pradesh and Karnataka during 2021–2022 was USD 26.30 million and USD 22.86 million, respectively. Among the total loss, mortality loss was highest, with 92% (USD 24.18 million) and 85% (USD 19.23 million) losses in Karnataka and Andhra Pradesh, respectively. Similarly, the highest loss was in sheep compared to goats in both states. The weight loss, distress sale loss, treatment cost, and opportunity cost of labor are presented in Table 4.

### 3.6. PPR Vaccination Status

In Andhra Pradesh state, a total of 205 (76.8%) flocks were vaccinated against PPR, of which 113 flocks (113/158 = 71.5%) in were in the Ananthapur District and 92 flocks (92/109 = 84.4%) in the Prakasam District. It was also observed that 68.7% of animals in the surveyed flocks in the Ananthapur District and 88% of animals in the Prakasam District were vaccinated against PPR (Table 5). In Karnataka, a total of 212 (73.1%) flocks were vaccinated against PPR, of which 131 (131/160 = 81.9%) were in the Bellary District and 81 (81/130 = 62.3%) in the Mandya District. Among the surveyed flocks, 75.1% of sheep and goats in the Bellary District and 67.8% of small ruminants in the Mandya District were vaccinated. Generally, the vaccines and vaccination services are free during the mass vaccination campaign, but if farmers procure privately and vaccinate during other periods, the vaccine cost ranges between USD 7.25 to 7.63/per 100 doses, and the vaccination cost is USD 10.75 per 100 doses (Table 5).

### 3.7. PPR Vaccination Impact

**Figure 3:**
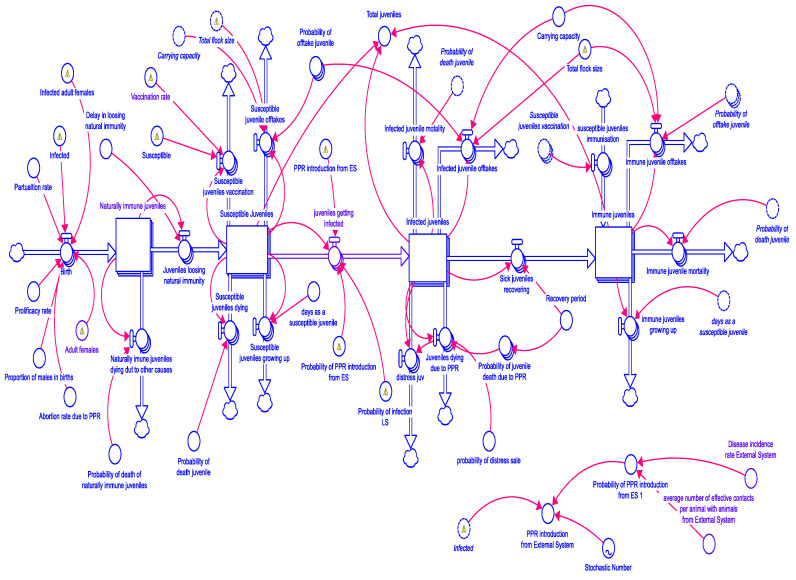
The integrated flock and disease dynamics module for juveniles (<6 months old) in Karnataka and Andhra Pradesh.

**Figure 4:**
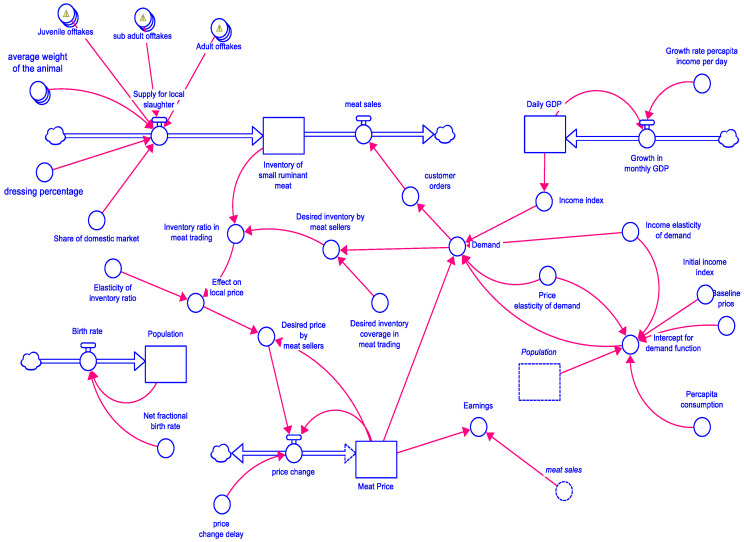
Marketing module in Karnataka and Andhra Pradesh.

**Figure 5:**
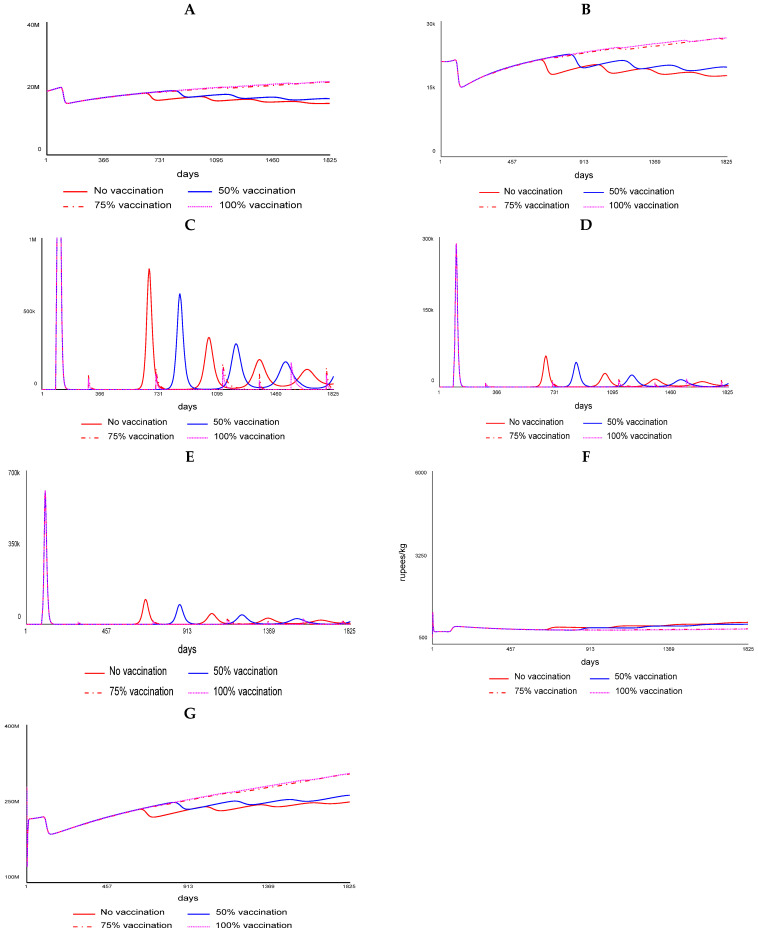
Impact of varying levels of PPR vaccination in Karnataka (Note: (**A**) change in flock size, (**B**) change in total offtake, (**C**) dynamics of PPR-infected population, (**D**) change in mortality, (**E**) change in recovery, (**F**) change in price effect, and (**G**) changes in overall earnings in Karnataka (projected for five years)).

**Figure 6:**
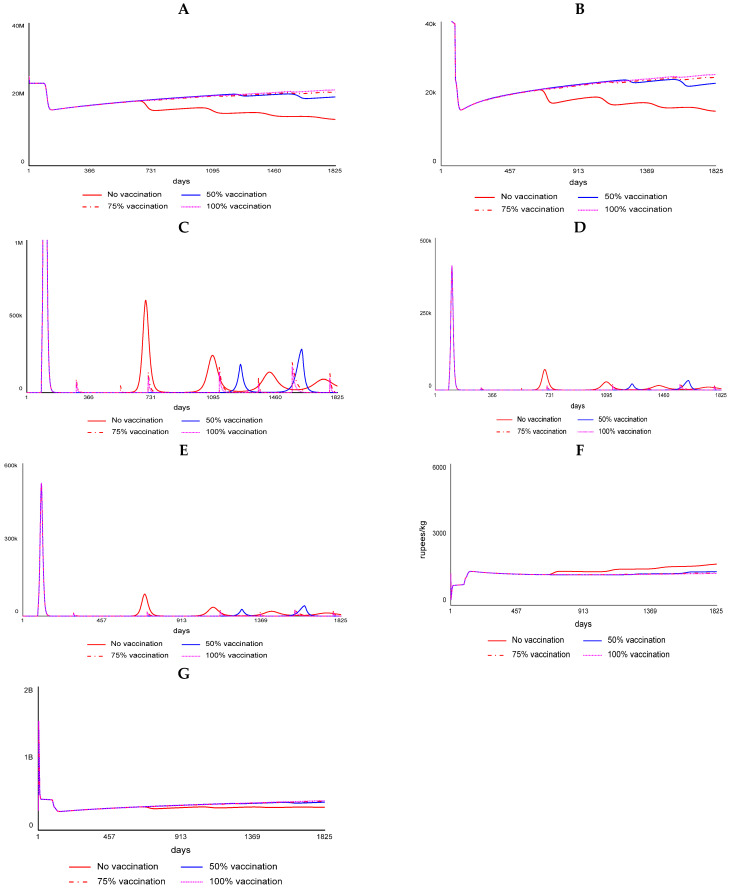
Impact of varying levels of PPR vaccination in Andhra Pradesh (Note: (**A**) change in flock size, (**B**) change in total offtake, (**C**) dynamics of PPR infected population, (**D**) change in mortality, (**E**) change in recovery, (**F**) change in price effect, and (**G**) changes in overall earnings in Andhra Pradesh (projected for five years)).

We ran the SD model for 5 years (1825 days) from 2020 to 2025. The PPR vaccination coverage-associated impacts like flock size, disease incidence, etc., in Karnataka (local system) was assessed, keeping the probability of disease spread from the contiguous Andhra Pradesh state (external system) at a fixed minimum of 5% incidence. Similarly, the impact of PPR vaccination in Andhra Pradesh (local system) vis-à-vis the disease spread from the neighboring state Karnataka (external system) was fixed at a minimum of 5% incidence. For the local system, the vaccination coverage scenarios assessed included no vaccination (0%), 50%, 75%, and 100%. The model framework for the juveniles (<6-month-old sheep/goats) is presented in Figure 3, and the marketing module is presented in Figure 4. Similar to juveniles, outcomes for sub-adults (6–12 months) and adults (>1 year) were also modeled. The modeling results showed that without vaccination, there is a huge wave of outbreaks, which diminishes over time in terms of the maximum number of infected animals each time an outbreak happens and increases in the size with the number of days it takes for the outbreak to pass (Figure 5 and Figure 6). Further, the series of outbreaks is much smaller with increased vaccination coverage. For each of the vaccination scenarios, the projected meat prices vary, implying the effect of meat supply on price. The results showed a systemic increase in flock size and offtakes and a decline in the number of infected cases and death cases under high vaccination coverage (75% and 100% coverage) compared to low-coverage scenarios.

## 4. Discussion

The present study aimed to understand the PPR burden under the mass vaccination implemented in two southern states of India, Andhra Pradesh and Karnataka, and to assess the impact of vaccination coverage in one state (local system) given the minimum possibility of infection from the other state (external system), as there is constant and recurring movement of animals for transit, trade, and grazing between these states with respective mass vaccination implementation. The modeling assumed two geographical regions: a local system (state) and external system (neighboring state). The transmission of PPR in the local system was modeled with two components: within-region transmission from interactions between susceptible and infected animals and cross-region transmission from the external system. This framework captures both internal and external influences on PPR outbreaks in the local system. 

In Karnataka and Andhra Pradesh, before mass vaccination implementation, the number of outbreaks/year was high (>150 outbreaks) [11,21,22], and it declined thereafter. This implies a significant reduction in PPR-related mortality and morbidity in these states after the implementation of mass vaccination. But, despite long years of mass vaccination in Karnataka from 2010–2011 and in Andhra Pradesh from 2007 (with some intermittent delay in some years due to administrative issues and other bottlenecks), PPR is still reported although in lesser magnitude. When we look at the states independently, the mass vaccination reduced the occurrence of outbreaks, but from a national perspective, decline in the contiguous states is needed simultaneously with zero outbreaks and elimination of virus circulation in the near term to reach the eradication stage. Given the constant movement of animals between the contiguous states in India and the variation in the time and geographical coverage of vaccination in different states, the impact of vaccination in one state needs to be studied vis-à-vis the probability of the infection from the neighboring states to understand the real impact of PPR vaccination.

The PPR incidence in the surveyed states was <4.0%, which is lower than the earlier reported >50% incidence [2], and 8% incidence [23] and 4.8 to 9.8% [24,25]. The lower incidence in the present study is due to the mass vaccination implementation in Andhra Pradesh since 2007 and in Karnataka from 2010–2011. Furthermore, CFR was considerably higher in animals <6 months of age than in older animals, and this is in line with the earlier literature [4]. In the present study, PPR infection in sheep was found to be higher than in goats, whereas in northern Indian states, the infection in goats was higher than in sheep [14] This might be due to the population density per se in the study region. In the northern states of India, the goat population density is higher, whereas in southern states like Andhra Pradesh and Karnataka, the sheep population is higher.

The estimated mortality loss, body weight reduction loss, distress sale loss, treatment cost, and the opportunity cost of labor per animal were in the ranges of USD 35.1–108.1 and USD 40.5–189.2; USD 3.4–11.5 and 4.1–16.2; USD 60.8–114.9 and 40.5–103.6; USD 0.3–4.5 and USD 0.3–3.4; and USD 0.2–5.1 and 0.2–3.0 in Andhra Pradesh and Karnataka states, respectively, which concurs with earlier reports [4,24]. The projected loss due to PPR in Andhra Pradesh and Karnataka was USD 26.30 million and USD 22.86 million, respectively, which is relatively less compared to earlier estimates of USD 653–669 million for the whole country, comprising 28 states and 8 union territories [8].

The present study also observed that 73 to 77% of flocks and 74 to 77% of the animals were vaccinated against PPR, indicating high vaccination coverage, and this might be the possible reason for the lower PPR incidence (<4%) during the survey period. The SD model results showed a systemic increase in flock size and offtakes and a decline in the number of infected cases and death cases under high vaccination coverage (75% and 100% coverage) scenarios compared to low-coverage scenarios. Higher vaccination coverage increases flock size and offtakes, which might reduce the price of meat, but considering the continuous demand increase due to increased income growth and the high human population growth in India, this might offset the fall in meat prices. The reduction in flock size and offtakes in Karnataka or Andhra Pradesh is lower, with the reduced number of contagious animals moving from one state to another implying high vaccination coverage in the neighboring states, benefitting other contiguous states; hence, coordinated interstate efforts in terms of timing of vaccinations, coverage of animals, and vaccination at the borders before entry into the neighboring state offer better prospects, as efforts in one state have positive externalities in the neighboring state through reduced disease incidence. Further, at the macro level, reduced PPR incidence due to coordinated vaccination among the contiguous states increases offtakes and reduces the price of meat. The reduced price might benefit poor households with better nutrition outcomes, and to some extent, the reduction in prices will be offset by increased demand due to population and income growth. The increased flock size due to reduced disease also needs support on the input front, especially increased fodder supply, to enhance the carrying capacity of the sheep and goats. The limitation of the model analysis is that it assumes uniformity within the system (local system) and focuses solely on interactions between the local and external systems, potentially overlooking other external factors, such as broader transmission dynamics beyond these two regions/systems. However, the model underwent rigorous validation with experts from Indian Council of Agricultural Research (ICAR) and external PPR specialists at multiple stages. First, data validation was conducted to ensure the accuracy and reliability of the input data. Second, the value chain mapping was reviewed to confirm the logical framework and relationships within the value chain. Finally, result validation was performed to verify the accuracy and robustness of the model’s outputs and conclusions.

## 5. Conclusions

As per the National Strategic Plan for Eradication of PPR by 2030, annual mass vaccination is planned in all states (including the study states) and also has legal back-up for the control and eradication of livestock disease. Despite vaccination since 2010, the sporadic outbreak reported in these states underscores the importance of timely vaccination within these contiguous regions and emphasizes the necessity of collaboration with neighboring states. The potential increase in flock size and offtakes due to PPR control through vaccination holds promise for boosting income among producers and various value chain actors. Coordinated inter-state vaccination efforts present a more promising strategy, as successful initiatives in one state can yield positive externalities, such as fewer outbreaks in neighboring states. This collaborative approach is increasingly crucial, especially considering the Government of India’s ambitious plan to eradicate the disease by 2030, aligning with the global eradication strategy.

## Figures and Tables

**Table 1:** General characteristics of sample farms in Andhra Pradesh and Karnataka.

Sl. No.	Particulars	Unit	Surveyed Districts					
			Andhra Pradesh			Karnataka		
			Ananthapur	Prakasam	Pooled	Bellary	Mandya	Pooled
1	No. of farms/flocks surveyed	No.	158	109	267	160	130	290
2	Age (median)	Years	42	49	45	43	48	46
3	Education							
	Illiterate	No.	113 (71.5)	79 (72.5)	192 (71.9)	118 (73.7)	93 (71.5)	211 (72.8)
	Primary (1st to 7th)	No.	30 (19)	21 (19.3)	51 (19.1)	29 (18.1)	21 (16.2)	50 (17.2)
	High school/secondary (8th to 10th)	No.	13 (8.2)	9 (8.2)	22 (8.2)	11 (6.9)	16 (12.3)	27 (9.3)
	Graduation and above	No.	2 (1.3)	0 (0)	2 (0.7)	2 (1.3)	0 (0)	2 (0.7)
4	Family size (median)	No.	4	6	6	5	5	5
5	Land holdings (acres)							
	Landless	No.	69 (43.7)	47 (43.1)	116 (43.4)	50 (31.3)	8 (6.1)	58 (20.0)
	Small (1 to <5)	No.	74 (46.8)	56 (51.4)	130 (48.7)	80 (50)	115 (88.5)	195 (67.2)
	Medium (5 to 10)	No.	11 (7.0)	6 (5.5)	17 (6.4)	17 (10.6)	7 (5.4)	24 (8.3)
	Large (>10)	No.	4(2.5)	0 (0)	4 (1.5)	13 (8.1)	0 (0)	13 (4.5)
6	Annual farm income (USD)							
	Very low (<625)	No.	25 (15.8)	8 (7.33)	33 (12.4)	18 (11.3)	11 (8.4)	29 (10)
	Low (626–1250)	No.	51 (32.3)	31 (28.44)	82 (30.7)	45 (28.1)	66 (50.8)	111 (38.3)
	Medium (1251–2500)	No.	55 (34.8)	55 (50.45)	110 (41.2)	49 (30.7)	52 (40)	101 (34.8)
	High (>2501)	No.	27(17.1)	15 (13.76)	42 (15.7)	48 (30)	1 (0.8)	49 (16.9)
7	Total no. of sheep and goats	No.	19,035	13,873	32,908	22,268	4892	27,160
8	Average no. of sheep and goats per farm	No.	120	127	123	139	38	94

Figures in parentheses indicate percentage to total.

**Table 2:** Distribution of mortality and morbidity in sheep and goats due to PPR in Karnataka and Andhra Pradesh.

Groups	Category	No. of Animals at Risk		PPR-Affected Animals (%)		No. of Death Cases (%)		CFR (%)	
		Andhra Pradesh	Karnataka	Andhra Pradesh	Karnataka	Andhra Pradesh	Karnataka	Andhra Pradesh	Karnataka
Species	Sheep	31,688	23,840	1036 (3.3)	917 (3.8)	864 (2.7)	602 (2.5)	83.4	65.6
	Goats	1220	3320	10 (0.8)	39 (1.2)	10 (0.8)	21 (0.6)	100.0	53.8
	Total	32,908	27,160	1046 (3.2)	956 (3.5)	874 (2.7)	623 (2.3)	83.6	65.2
	χ^2^			22.9 ***	60.5 ***	15.8 ***	45.7 ***		
Sheep									
Age-wise	<6 months	7363	5126	769 (10.4)	458 (8.9)	662 (9)	417 (8.1)	86.1	91.0
	6–12 months	11,621	8378	177 (1.5)	239 (2.9)	131 (1.1)	99 (1.2)	74.0	41.4
	>1 year	12,704	10,336	90 (0.7)	220 (2.1)	71 (0.6)	86 (0.8)	78.9	39.1
	χ^2^			1574.1 ***	463.7 ***	1426.6 ***	837.2 ***		
Sex-wise	Male	5042	3157	328 (6.5)	243 (7.7)	278 (5.5)	220 (7)	84.8	90.5
	Female	26,646	20,683	708 (2.7)	674 (3.3)	586 (2.2)	382 (1.8)	82.8	56.7
	χ^2^			197.3 ***	144.7 ***	174.4 ***	289.8 ***		
Goats									
Age-wise	<6 months	343	729	8 (2.3)	24 (3.3)	8 (2.3)	16 (2.2)	100.0	66.7
	6–12 months	428	954	2 (0.5)	5 (0.5)	2 (0.5)	2 (0.2)	100.0	40.0
	>1 year	449	1637	0 (0)	10 (0.6)	0 (0)	3 (0.2)	0	30.0
	χ^2^			14.1 ***	36.1 ***	14.1 ***	36.3 ***		
Sex-wise	Male	217	637	0 (0)	9 (1.4)	0 (0)	7 (1.1)	0	77.8
	Female	1003	2683	10 (1)	30 (1.1)	10 (1)	14 (0.5)	100.0	46.7
	χ^2^			1.12 ^NS^	0.17 ^NS^	1.13 ^NS^	1.9 ^NS^		
Pooled									
Age-wise	<6 months	7706	5855	777 (10.1)	482 (8.2)	670 (8.7)	433 (7.4)	86.2	89.8
	6–12 months	12,049	9332	179 (1.5)	244 (2.6)	133 (1.1)	101 (1.1)	74.3	41.4
	>1 year	13,153	11,973	90 (0.7)	230 (1.9)	71 (0.5)	89 (0.7)	78.9	38.7
	χ^2^			1571.8 ***	495.5 ***	1427.1 ***	869.5 ***		
Sex-wise	Male	5259	3794	328 (6.2)	252 (6.6)	278 (5.3)	227 (6)	84.8	90.1
	Female	27,649	23,366	718 (2.6)	704 (3)	596 (2.2)	396 (1.7)	83.0	56.3
	χ^2^			189.1 ***	125.5 ***	166.3 ***	265.9 ***		

Figures in parentheses represent percentage. CFR = case fatality rate. ***, significant at 1%; NS = non-significant.

**Table 3:** Estimated median loss per animal (USD) due to PPR in sheep and goats in Andhra Pradesh and Karnataka at 2021–2022 prices.

Loss Components (USD)	Sheep		Goats		Pooled	
	Andhra Pradesh	Karnataka	Andhra Pradesh	Karnataka	Andhra Pradesh	Karnataka
Mortality	54.6 (2, 35.1–108.1)	58.3 (4.2, 40.5–189.2)	59.2 (4.9, 40.5–81.1)	65.7 (9.4, 47.3–94.6)	54.6 (4.9, 35.1–108.1)	63.6 (3.9, 40.5–189.2)
Body weight reduction	5.4 (0.2, 3.4–11.5)	8.6 (0.4, 4.1–16.2)	0 (0, 0–0)	10.9 (1.1, 6.5–12.2)	5.4 (0.2, 3.4–11.5)	12.2 (0.4, 4.1–16.2)
Distress sale	71 (28.1, 60.8–114.9)	56.4 (9.1, 40.5–103.6)	0 (0, 0–0)	0 (0, 0–0)	71 (28.1, 60.8–114.9)	56.4 (9.1, 40.5–103.6)
Treatment cost	0.9 (0.1, 0.3–2.6)	1.2 (0.1, 0.3–3.4)	0.9 (0.1, 0.3–4.5)	1.2 (0.1, 0.9–2.7)	0.9 (0.1, 0.3–4.5)	1.2 (0.1, 0.3–3.4)
Opportunity cost of labor	1.1 (0.1, 0.2–2.3)	1.2 (0.1, 0.2–2.7)	1.1 (0.1, 0.3–5.1)	1.2 (0.1, 0.2–1.5)	1.1 (0.1, 0.2–5.1)	1.2 (0.1, 0.2–3)

Figures in parentheses represent standard error and range values.

**Table 4:** Projected loss in sheep and goats in Andhra Pradesh and Karnataka at 2021–2022 prices (in USD million).

States	Disease Incidence DI (%)	Species	Loss Components					Total Projected Loss/Year (a + b + c + d + e)
			Mortality (a)	Weight Loss (b)	Distress Sale (c)	Treatment Cost (d)	Opportunity Cost of Labor (e)	
Andhra Pradesh	3.3	Sheep	22.75 (91.8)	0.43 (1.7)	0.38 (1.5)	0.54 (2.2)	0.68 (2.7)	24.78 (100)
	0.8	Goat	1.43 (94.1)	0.0 (0)	0.0 (0)	0.04 (2.6)	0.05 (3.3)	1.52 (100)
		Over all	24.18 (91.9)	0.43 (1.6)	0.38 (1.4)	0.58 (2.2)	0.73 (2.8)	26.30 (100)
Karnataka	3.8	Sheep	16.94 (84.5)	1.23 (6.1)	0.91 (4.5)	0.48 (2.4)	0.48 (2.4)	20.04 (100)
	1.2	Goat	2.30 (81.3)	0.35 (12.4)	0.0 (0)	0.09 (3.2)	0.09 (3.2)	2.83 (100)
		Over all	19.23 (84.1)	1.58 (6.9)	0.91 (4.0)	0.57 (2.5)	0.57 (2.5)	22.86 (100)

Figures in parentheses indicate percent of total projected loss.

**Table 5:** PPR vaccination levels among the sample farmers in the surveyed districts of the states of Karnataka and Andhra Pradesh during 2019–2020 and 2020–2021.

Particulars	Andhra Pradesh			Karnataka		
	Ananthapur District	Prakasam District	Pooled	Bellary District	Mandya District	Pooled
Total farms surveyed	158	109	267	160	130	290
Total farms vaccinated	113 (71.5)	92 (84.4)	205 (76.8)	131 (81.9)	81 (62.3)	212 (73.1)
Total farms not vaccinated	45 (28.5)	17 (15.6)	62 (23.2)	29 (18.1)	49 (37.7)	78 (26.9)
Total sheep and goats in the farms	19,035	13,873	32,908	22,268	4892	27,160
Total sheep and goats vaccinated	13,079 (68.7)	12,204 (88)	25,283 (76.8)	16,725 (75.1)	3317 (67.8)	20,042 (73.8)
Total sheep and goats not vaccinated	5956 (31.3)	1669 (12)	7625 (23.2)	5543 (24.9)	1575 (32.2)	7118 (26.2)
Average cost of vaccine (in USD for 100 doses) if procured by the farmer			7.63			7.25
Average cost of vaccination (in USD per 100 doses) if privately vaccinated	10.75	free of cost	10.75	free of cost	free of cost	free of cost

Note: Figures in the parentheses indicate percentage of total.

## Data Availability

The primary data collected and analyzed during this study are available from the corresponding author on reasonable request.

## Supplementary Materials

The following supporting information can be downloaded at: https://www.mdpi.com/article/10.3390/v17010023/s1, Table S1: Variables used in the study and the source of information. References [4,26,27] are cited in the Supplementary Materials.
