# Supplementary material for: Assessment of Vaccination Impact in PPR-Control Program Implemented in Southern States of India: A System Dynamics Model Approach
Source: Viruses. 2024 Dec 27;17(1):23. doi: 10.3390/v17010023 (PMC11769144; doi:10.3390/v17010023)
Supplement: Supplementary file 1 [file viruses-17-00023-s001.zip › viruses-3330550-supplementary.pdf]

Supplementary Table S1. Variables used in the study and the source of information

**Parameters**	**Reference/ source**
**Socio-economic parameters**	
Age, education, income, family size, land holding	Primary survey
**Sheep and goat population inventory and animal production characteristics**	
< 6 months-juvenile male	Livestock census, 2019 (https://cdnbbsr.s3waas.gov.in/s369dafe8b58066478aea48f3d0f384820/uploads/2024/02/202402131737819886.pdf)
6-12 month-sub-adult male	Livestock census, 2019 (https://cdnbbsr.s3waas.gov.in/s369dafe8b58066478aea48f3d0f384820/uploads/2024/02/202402131737819886.pdf)
>1 year-adult male	Livestock census, 2019 (https://cdnbbsr.s3waas.gov.in/s369dafe8b58066478aea48f3d0f384820/uploads/2024/02/202402131737819886.pdf)
< 6 month-juvenile female	Livestock census, 2019 (https://cdnbbsr.s3waas.gov.in/s369dafe8b58066478aea48f3d0f384820/uploads/2024/02/202402131737819886.pdf)
6-12 month- sub-adult female	Livestock census, 2019 (https://cdnbbsr.s3waas.gov.in/s369dafe8b58066478aea48f3d0f384820/uploads/2024/02/202402131737819886.pdf)
>1 year- adult female	Livestock census, 2019 (https://cdnbbsr.s3waas.gov.in/s369dafe8b58066478aea48f3d0f384820/uploads/2024/02/202402131737819886.pdf)
Parturition rate per year	Expert opinion
Prolificacy rate	Expert opinion
Rate of male female ratio at birth	Expert opinion
Abortion rate	Expert opinion
**Offtake of animals per year (proportion)**	
< 6 months-juvenile male	Primary survey
6-12 month-sub-adult male	Primary survey

>1 year-adult male	Primary survey
< 6 month-juvenile female	Primary survey
6-12 month- sub-adult female	Primary survey
>1 year- adult female	Primary survey
**Average weight of the healthy animals (kgs)**	
< 6 months-juvenile male	Weighed during the survey
6-12 month-sub-adult male	Weighed during the survey
>1 year-adult male	Weighed during the survey
< 6 month-juvenile female	Weighed during the survey
6-12 month- sub-adult female	Weighed during the survey
>1 year- adult female	Weighed during the survey
**PPR incidence rate**	
< 6 months-juvenile male	Primary survey
6-12 month-sub-adult male	Primary survey
>1 year-adult male	Primary survey
< 6 month-juvenile female	Primary survey
6-12 month- sub-adult female	Primary survey
>1 year- adult female	Primary survey
**Mortality rate due to PPR**	
< 6 months-juvenile male	Primary survey
6-12 month-sub-adult male	Primary survey
>1 year-adult male	Primary survey
< 6 month-juvenile female	Primary survey
6-12 month- sub-adult female	Primary survey
>1 year- adult female	Primary survey
**Case fatality rate**	
< 6 months-juvenile male	Primary survey
6-12 month-sub-adult male	Primary survey
>1 year-adult male	Primary survey
< 6 month-juvenile female	Primary survey
6-12 month- sub-adult female	Primary survey
>1 year- adult female	Primary survey
**Value (USD/animal)**	
< 6 months-juvenile male	Primary survey

6-12 month-sub-adult male	Primary survey
>1 year-adult male	Primary survey
< 6 month-juvenile female	Primary survey
6-12 month- sub-adult female	Primary survey
>1 year- adult female	Primary survey
**Epidemiological parameters**	
Naturally immune population proportion in different age groups	Expert opinion
Delay in loosing natural immunity	Expert opinion
Average duration of infection of PPR in farm	Primary survey
Number of animals vaccinated	Primary survey
Carrying capacity	Expert opinion
Vaccine cost per animal	Primary survey
Vaccination cost	[4]
**Recovery time for the infected animals (days)**	
< 6 months-juvenile male	Primary survey
6-12 month-sub-adult male	Primary survey
>1 year-adult male	Primary survey
< 6 month-juvenile female	Primary survey
6-12 month- sub-adult female	Primary survey
>1 year- adult female	Primary survey
**Marketing variables**	
Price of meat/kg	Primary survey
Per capita consumption	[26]
Share of domestic market	Expert opinion
Price change delay	Expert opinion
Income elasticity of demand	[27]
Price elasticity of demand	https://books.google.com.au/books/about/Agricultural_Product_Prices.html?id=DLszAAAAMAAJ&redir_esc=y
